# Investigation of functional connectivity differences based on anxiety tendencies

**DOI:** 10.3389/fnbeh.2024.1498612

**Published:** 2024-12-10

**Authors:** Misuzu Oishi, Noriko Sakurai, Yuki Kawasaki, Kei Sasaki, Satoshi Kasai, Naoki Kodama

**Affiliations:** ^1^Graduate School of Health and Welfare, Niigata University of Health and Welfare, Niigata, Japan; ^2^Department of Radiological Technology, Niigata University of Health and Welfare, Niigata, Japan

**Keywords:** resting-state functional MRI, functional connectivity, salience network, anterior cingulate cortex, anterior insula, anxiety

## Abstract

**Introduction:**

Anxiety is an emotion necessary for human survival. However, persistent and excessive anxiety can be clinically challenging. Increased anxiety affects daily life and requires early detection and intervention. Therefore, a better understanding of the neural basis of mild anxiety is needed. However, previous studies have focused primarily on resting-state functional magnetic resonance imaging (rs-fMRI) in patients with psychiatric disorders presenting with anxiety. Notably, only a few studies have been conducted on healthy participants, and the relationship between anxiety and functional brain connectivity in the healthy range remains unclear. Therefore, in this study, we aimed to clarify the differences in functional brain connectivity at different degrees of anxiety among healthy participants.

**Methods:**

This study included 48 healthy participants with no history of psychiatric disorders. Participants were administered The General Health Questionnaire (GHQ) 60, a psychological test for assessing anxiety, and the Manifest Anxiety Scale (MAS). The participants then underwent rs-fMRI. Based on the results of each psychological test, the participants were classified into normal and anxiety groups, and the functional connectivity between the two groups was compared using a seed-to-voxel analysis.

**Results:**

Comparison of functional brain connectivity between the normal and anxiety groups classified based on the GHQ60 and MAS revealed differences between brain regions comprising the salience network (SN) in both psychological tests. For the GHQ60, the anxiety group showed reduced connectivity between the right supramarginal gyrus and insular cortex compared with the normal group. However, for the MAS, the anxiety group showed reduced connectivity between the right supramarginal and anterior cingulate cortical gyri compared with the normal group.

**Conclusion:**

Functional connectivity within the SN was reduced in the group with higher anxiety when functional brain connectivity at different anxiety levels was examined in healthy participants. This suggests that anxiety is involved in changes in the functional brain connectivity associated with emotional processing and cognitive control.

## 1 Introduction

Anxiety is an emotion caused by anticipating a real or imagined future threat or danger (Penninx et al., 2021). Anxiety is an essential emotion for living. For example, anxiety constitutes an alert or warning mechanism necessary to make the best choices in dangerous situations, thus improving survival probability (Gutiérrez-García and Contreras, 2013). However, increased, severe, or persistent anxiety compared with a threat requires clinical attention (Penninx et al., 2021). Increased anxiety that prevents a person from leading a normal life is considered an anxiety disorder, and this requires treatment (Ströhle et al., 2018). Anxiety disorders often develop early in life, such as in childhood, adolescence, or early adulthood, and can be chronic, persistent, or recurrent throughout life (Ströhle et al., 2018; Goddard, 2017). In addition, anxiety disorders have various adverse physical effects and are associated with an increased risk of cardiovascular disease and the development of respiratory and gastrointestinal disorders (Peng et al., 2024; Sareen et al., 2006). Therefore, early intervention is necessary because anxiety disorders affect both social and daily lives. Thus, early interventions such as cognitive-behavioral therapy can reduce mild anxiety and may even prevent the onset of anxiety disorders (Feldner and Zvolensky, 2006).

Studies using task-based functional magnetic resonance imaging (fMRI), which induces anxiety, have reported the activation of regions such as the amygdala, insular cortex, prefrontal cortex, anterior cingulate cortex, and hippocampus (Etkin and Wager, 2007; Shin and Liberzon, 2010). In contrast, psychiatric disorders are characterized by structural and functional abnormalities in multiple brain regions. This indicates that activation patterns in specific brain regions alone are insufficient to understand the neural basis of anxiety; therefore, they must be assessed at the network level (Menon, 2011). Brain networks are investigated using resting-state fMRI (rs-fMRI) to determine the functional connections between brain regions. rs-fMRI is a relatively new method for defining brain networks at the macro level by examining synchronous blood-oxygen-level-dependent (BOLD) signal variations between brain regions measured at rest (Fox and Raichle, 2007). Typical examples of such networks are the default mode network (DMN), frontoparietal network (FPN), and salience network (SN). The interaction among these three networks is involved in almost all cognitive functions (Zhang et al., 2015). The DMN increases activity during mental exploration of oneself, such as autobiographical memory, examining social interactions, and thinking about one’s future (Buckner et al., 2008). The FPN, also known as the central executive network, exhibits increased activity during cognitively demanding tasks (Goulden et al., 2014). For example, in problem-solving and goal-directed behavior, decisions are made by maintaining and processing information in the working memory (Menon, 2011). As a relationship between these two networks, the SN is responsible for switching between the FPN and the DMN (Palaniyappan and Liddle, 2012). The SN is involved in sensory and reflective thinking and is a hub for integrating information about goals and plans. Therefore, psychiatric and neurological disorders can be understood by examining DMN, FPN, and SN dysfunctions (Menon, 2011). Decreased connectivity of the ventral and dorsal medial prefrontal cortices, which comprise the DMN, has been reported in patients with generalized anxiety disorders. These two sites are the primary sites in the DMN involved in emotional and self-referential processing. Patients with generalized anxiety disorders are said to have impaired processing of these functions (Steinhäuser et al., 2023).

Regarding studies on individuals with healthy range anxiety, reduced connectivity between the insular and dorsal anterior cingulate cortices, comprising the SN, has been reported in healthy participants with high trait anxiety. Notably, this reduced functional connectivity reflects reduced cognitive control (Geng et al., 2015). The insular and dorsal prefrontal cortices are part of the prefrontal cortex, suggesting that trait anxiety is associated with decreased activity in the attentional control mechanisms of the prefrontal cortex (Bishop, 2009).

To date, rs-fMRI studies have mainly been conducted in patients with psychiatric disorders. However, few studies have been conducted on healthy range anxiety, and the relationship between healthy range anxiety and functional brain connectivity remains unclear. Additionally, many studies if any, target only trait anxiety, which is personality anxiety. Therefore, this study considered trait anxiety as well as the degree of anxiety and assessed the true state of anxiety more comprehensively using The General Health Questionnaire (GHQ) 60 and Manifest Anxiety Scale (MAS). The GHQ60 examines the degree and extent of anxiety by assessing current mental functioning and new stressors (Goldberg et al., 1985). In contrast, the MAS examines the characteristics of long-term anxiety and its degree (Hathaway et al., 2013). Therefore, the GHQ60 and MAS can be used to evaluate acute and chronic anxiety states in healthy individuals.

Individuals with high healthy range anxiety exhibit decreased SN connectivity, whereas those with anxiety disorders show decreased DMN connectivity. Therefore, in this study, we hypothesized that the functional connectivity of the SN would decrease in the high anxiety state in the healthy range, and beyond that, the functional connectivity of the DMN would also decrease. Therefore, we aimed to clarify the differences in functional brain connectivity in different anxiety levels among healthy participants. Functional changes in the brain precede the onset of anxiety disorders (Strawn et al., 2021). Therefore, understanding changes in functional brain connectivity in individuals with high anxiety in the healthy range may lead to the early detection of mental disorders and early intervention. Furthermore, this is the first study to examine the relationship between the healthy range of anxiety and functional connectivity using the GHQ60 and MAS.

## 2 Materials and methods

### 2.1 Participants

Forty-eight participants (26 males and 22 females, aged 20.3 ± 1.3 years) were included in the study. Patients with a history of psychiatric disorders and those with contraindications to MRI examinations were excluded. The female subjects in this study considered the effects of premenstrual syndrome. Gudipally and Sharma (2023) reported that 80–90% of women may experience anxiety and physical pain from 1 week before the start of menstruation to a few days after the start of menstruation. In order to exclude the impact of psychological and physiological fluctuations related to the menstrual cycle on the research results, the period from 1 week before the expected start of menstruation to the end of menstruation was excluded from the subjects. The study was explained to the participants, and their written consent was obtained. This study was approved by the Research Ethics Committee of the Niigata University of Health and Welfare (approval No. 19113-230707).

### 2.2 Psychological testing

Participants were classified into normal and anxiety groups based on their GHQ60 and MAS results. The GHQ60 and MAS are used as screening tests for undiagnosed mental illnesses. GHQ60 is an effective psychological test for understanding, evaluating, and detecting neurotic symptoms. The GHQ60 examines the participant’s condition 2–3 weeks before presentation. It GHQ60 assesses whether healthy mental functioning can be maintained or if there are new events causing distress to the examinee and does not consider the examinee’s characteristics. The GHQ60 is based on the GHQ scoring method. The GHQ scoring method sums up the total number of the “bad” or “very bad” answers of four options. Notably, participants with GHQ scores of 0–16 points were considered the healthy group, and those with scores of 17–60 points were considered the anxiety group. The GHQ score of 16/17 is the preferred classification point for conducting mental health surveys in research on populations (Goldberg et al., 1985).

The MAS is a psychological test designed to measure the perceived physical and mental anxiety experienced by individuals and clarify the degree of such anxiety. The MAS assesses trait anxiety; scores do not change with situational changes, such as relaxation or pressure interviews (Hathaway et al., 2013). The Minnesota Multiphasic Personality Inventory (MMPI) scoring method was used to determine MAS scores. The MMPI method is a two-case method in which no points are given for “do not know” responses to questions, and only one point is given for “yes” or “no” responses. A method that gives 0.5 points for “I do not know” is also available; however, it is not recommended due to the uncertainty in the accuracy of the response (Hathaway et al., 2013). In the MAS, following the scoring grading criteria for college students, participants with anxiety scores of 0–22 points were considered the healthy group, and those with 23–50 points were considered the anxiety group.

### 2.3 Image acquisition

MRI data were collected using a 3 T Vantage Galan MRI scanner (Canon Medical Systems, Tochigi, Japan) with a 32-channel head coil. T1-weighted images were used as structural images. T1-weighted images were acquired using a magnetization-prepared rapid gradient-echo sequence with the following imaging parameters: repetition time (TR) = 5.8 ms, echo time (TE) = 2.7 ms, inversion time (TI) = 900 ms, flip angle (FA) = 9°, matrix size = 256 × 256, field of view (FOV) = 230 × 230 mm, and slice thickness = 1.2 mm. Structural imaging was performed using fMRI imaging. The participants were instructed to rest with their eyes open during fMRI imaging. The echo planar imaging sequence was used for fMRI, and the imaging conditions were TR = 2.0 ms, TE = 25 ms, FA = 85°, number of matrices=64 × 64, FOV = 240 × 240 mm, and slice thickness = 3 mm. The imaging time was 5 min, and the room was dark during the MRI data collection.

### 2.4 Data analysis

Statistical Parametric Mapping 12 running on MATLAB (MathWorks, Natick, MA, United States) and the CONN functional connectivity toolbox v.17 were used for data analysis. Functional connectivity was analyzed in the following order: spatial pretreatment, time-series pretreatment, individual analysis, and population analysis. Spatial preprocessing included slice-timing correction, body-motion correction, spatial standardization, and spatial smoothing. Large head movements exceeding the 95th percentile were considered outliers for the body motion correction. Scans identified as outliers were excluded from the analysis. For spatial normalization, the images were standardized to the Montreal Neurological Institute standard brain corresponding to the International Consortium for Brain Mapping 152 template to eliminate individual differences in brain shape. Spatial smoothing was performed using a three-dimensional Gaussian filter with a half-width of 8 mm to mitigate the image noise and individual brain differences caused by the series of processing. For time-series preprocessing, a bandpass filter was applied in the frequency range of 0.008–0.009 Hz to remove artifacts, such as body motion and physiological noise.

However, for individual analysis, a seed-to-voxel analysis was performed; the time-series data in the seed region were the average of the time-series data of the BOLD signal in all voxels in the region of interest (ROI). The location of the ROI, the seed region, was defined using the template coordinates in the CONN. Correlations of time-series data were obtained between the voxels in the seed and whole brain regions. In addition, the Pearson’s correlation coefficients obtained were transformed by Fisher’s Z. The functional connectivity of the major ROIs and voxels comprising each network was determined for the DMN, SN, FPN, dorsal attention, visual, language, sensorimotor, and cerebellar networks. In the population analysis, functional coupling was compared by subtracting the functional coupling of the normal group from that of the anxiety groups, which were classified based on each psychological test. The significance level was set at 5%.

### 2.5 Statistical analysis

Chi-square tests and Welch’s t-tests were used to statistically analyze the participant data and psychological test scores. Chi-square tests were used to analyze differences in the sex composition of the normal and anxiety groups. Welch’s t-tests were used to analyze differences in age and psychological test scores. Statistical significance was set at *p* < 0.05.

For networks with significantly altered functional connectivity, we analyzed the correlation between psychometric test scores and Z-scores representing functional connectivity. The Z-score is the Z-transformed value of Pearson’s correlation coefficient obtained from the time-series data of synchronized BOLD signals between two ROIs. Correlation coefficients were evaluated using IBM SPSS Statistics Version 27 and Spearman’s rank correlation coefficient. Statistical significance was set at *p* < 0.05.

## 3 Results

### 3.1 Demographics and psychological tests

Table 1 shows the participant data and results of the psychological tests. For the GHQ60, 35 and 13 participants were in the normal and anxiety groups, respectively; however, for the MAS, 27 and 21 were in the normal and anxiety groups, respectively. There were no significant differences in sex composition and age between the normal and anxiety groups for either the GHQ60 or MAS. However, psychological test scores were significantly higher in the anxiety group than in the normal group for the GHQ60 and MAS.

**Table 1:** Participant data and the results of the psychological tests.

		Normal group	Anxiety group	*p*-value
GHQ60	Sex (male/female)	19/16	6/7	0.616
	Age (mean ± SD)	20.3 ± 1.42	20.3 ± 1.03	0.907
	Scores for each group (mean ± SD)	6.49 ± 4.72	13.1 ± 5.26	<0.001
	Overall score (mean ± SD)	11.7 ± 8.52	–	
MAS	Sex (male/female)	14/13	11/10	0.971
	Age (mean ± SD)	20.2 ± 0.980	20.3 ± 1.54	0.698
	Scores for each group (mean ± SD)	22.8 ± 4.00	28.0 ± 3.92	<0.001
	Overall score (mean ± SD)	19.6 ± 8.87	–	

### 3.2 rs-fMRI

**Figure 1:**
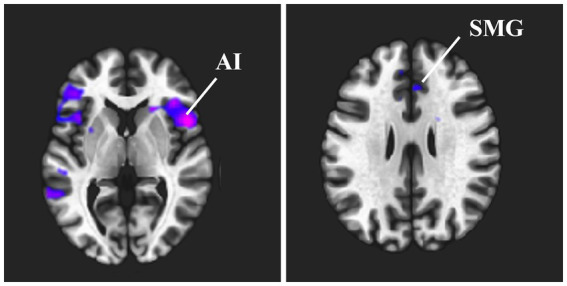
Brain regions that showed differences in functional connectivity between the normal and anxiety groups. This indicates the brain regions where a significant difference in functional connectivity strength was observed between the normal and anxiety groups when the right supramarginal gyrus was used as the seed region. Cold clusters indicate lower functional connectivity in the anxiety group than in the normal group. **(A)** Functional connectivity between the right supramarginal gyrus and the right insular cortex was reduced when the GHQ60 was used. **(B)** Functional connectivity between the right supramarginal gyrus and the anterior cingulate cortex was reduced when MAS was used. Abbreviation: ACC, anterior cingulate cortex; GHQ60, General Health Questionnaire 60; MAS, Manifest Anxiety Scale.

Figure 1 and Table 2 show the brain regions with differences in functional connectivity between the normal and anxiety groups. When comparing the functional connectivity of the brain between the normal and anxiety groups as classified based on the GHQ60 and MAS, differences were observed between the brain regions that constitute the SN in both psychological tests. When the GHQ60 was used, the anxiety group showed reduced connectivity between the right supramarginal gyrus and right insular cortex compared with the normal group. However, for MAS, the anxiety group showed reduced connectivity between the right supramarginal and anterior cingulate gyri compared with the normal group. Conversely, both groups showed no significant differences in functional connectivity in networks other than the SN, such as the DMN.

**Table 2:** Brain regions that showed differences in functional connectivity between the normal and anxiety groups.

Seed region	Cluster	MNI coordinate (mm)			*T* value	*p-*value
		x	y	z		
Right supramarginal gyrus	Right insula	50	12	2	−2.83	0.002
Right supramarginal gyrus	Anterior cingulate cortex.	2	20	48	−2.94	<0.001

### 3.3 Correlation analysis

**Figure 2:**
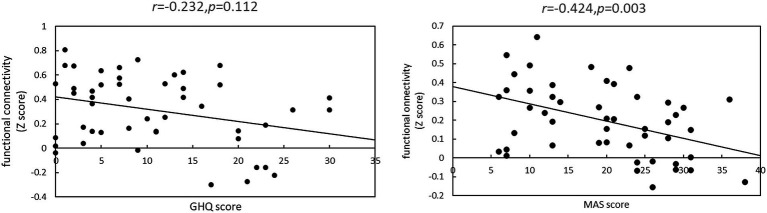
Relationship between each psychometric test psychological test score and functional connectivity. This represents correlations between Z-scores and psychological test scores between brain regions with significantly different functional connectivity strengths between the normal and anxiety groups. **(A)** There was no significant correlation between the strength of functional connectivity of the right supramarginal gyrus and right insular cortex and the GHQ60 scores (r = −0.232, *p* = 0.112). **(B)** There was a significant negative correlation between the strength of functional connectivity of the right superior marginal gyrus and anterior cingulate cortex and MAS scores (r = −0.424, *p* = 0.003).

Figure 2 shows the relationship between each psychological test score and functional connectivity.

When the GHQ60 was used, there was no significant correlation between psychological test scores and the Z-score of functional connectivity (r = −0.232, *p* = 0.112). However, when the MAS was used, there was a significant negative correlation between psychological test scores and Z-scores (r = −0.424, *p* = 0.003).

## 4 Discussion

Regarding the differences in brain functional connectivity associated with variations in anxiety among healthy participants, the GHQ60 and MAS revealed a decrease in connectivity between the brain regions constituting the SN in the anxiety groups. The SN plays a central role in cognitive control by integrating sensory inputs, directing attention, and activating the appropriate functional brain connections by focusing attention on stimuli of high importance (Peters et al., 2016). However, when the SN integrity is compromised, social, emotional, attentional, and cognitive control processes are impaired (Uddin, 2015). Notably, abnormalities in the structure and function of the anterior cingulate and insular cortices, critical regions of the SN, have been observed as a common neurobiological basis in various mental disorders (Peters et al., 2016). For example, patients with intense anxiety, such as schizophrenia and generalized anxiety disorder, have been reported to have reduced functional connectivity within the SN (Manoliu et al., 2013; Etkin et al., 2009).

Functional connectivity between the right supramarginal gyrus and right insular cortex was reduced in the anxiety group compared with the normal group when the GHQ60 was used. The insular cortex is involved in various cognitive, emotional, and regulatory functions, such as proprioceptive sensation, emotional response, and empathy. It acts as an integrating hub that links a strong network of brain regions responsible for emotional and cognitive functions (Menon and Uddin, 2010; Gogolla, 2017). Furthermore, Terasawa et al. (2013) reported that the right insular cortex is involved in neuroticism, extraversion, and agreeableness. In addition, the right supramarginal gyrus is associated with emotion recognition (Wada et al., 2021). In a study using rs-fMRI, Hu et al. (2019) reported that patients with depression have reduced connectivity between the right supramarginal gyrus and right insular cortex.

Moreover, Zhang et al. (2021) reported that decreased connectivity between the right supramarginal gyrus and insular cortex is associated with the cause of depression. Depressive symptoms, which are typical symptoms of depression, result from inappropriate emotion regulation, which is a failure to regulate mood and emotions caused by a lack of cognitive control (Kökönyei et al., 2024; Joormann and Gotlib, 2010). Consequently, reduced connectivity between the right supramarginal gyrus and the right insular cortex within the SN, which is responsible for cognitive control, may be responsible for anxiety and mental illness. The GHQ60 used in this study is a psychological test that assesses short-term anxiety and stress and screens for neurosis based on the most recent mental situation. Liu et al. (2020) reported a positive correlation between neurotic tendencies and the degree of depressive symptoms. Therefore, decreased connectivity between the right insular cortex and the right supramarginal gyrus, which is associated with depression, was observed in the GHQ60 anxiety group, which is prone to neurosis.

In contrast, the GHQ60 scores and Z-scores for functional connectivity showed a trend toward a negative correlation; however, this was not significant. This may be because the effect of transient anxiety on functional connectivity detected using the GHQ60 was temporary, and the correlation between the GHQ60 score and Z-scores was weak. In addition, there were few participants in the anxiety group, which may have reduced the statistical power and made it difficult to detect a correlation.

The MAS decreased the connectivity between the right supramarginal gyrus and the anterior cingulate cortex in the anxiety group compared with the normal group. Gellci et al. (2019) reported that children and adolescents growing up in impoverished areas had reduced functional connectivity of the anterior cingulate cortex and supramarginal gyrus. Growing up in poverty is often stressful and makes people more susceptible to negative emotional information, making it difficult for them to suppress their sad feelings (Capistrano et al., 2016). This reduced functional connectivity between the anterior cingulate cortex and supramarginal gyrus shows that the decreased connectivity efficiency of the supramarginal gyrus network negatively affects cognition and emotion (Gellci et al., 2019). Furthermore, Yang et al. (2018) reported that reduced functional connectivity associated with the supramarginal gyrus increases vulnerability to negative emotions such as anxiety. In addition, the anterior cingulate cortex is closely involved with emotion, performing central functions of intellectual behavior, such as emotional self-regulation, problem-solving, self-awareness of errors, and adaptation to changing circumstances (Allman et al., 2001).

Furthermore, the anterior cingulate cortex is part of the network associated with negative emotions and pain. It is involved in cognitive control processes that optimize behavior in response to negative emotional and pain stimuli (Tolomeo et al., 2016). This suggests reduced functional connectivity between the anterior cingulate cortex and supramarginal gyrus increases anxiety. Therefore, the reduced connectivity between the right supramarginal gyrus and anterior cingulate cortex may reflect the persistence of negative value judgments, likely due to impaired emotion regulation resulting from a tendency toward negative emotions. Furthermore, the MAS used in this study is a psychological test that assesses trait anxiety rather than state anxiety. Sep et al. (2019) reported that individuals with high-trait anxiety are frequently in a state of anxiety, and negative emotions are induced. Therefore, decreased connectivity between the right supramarginal gyrus and the anterior cingulate cortex was observed in the MAS-anxiety group, which tended to have stronger trait anxiety.

Additionally, there was a significant negative correlation between the MAS scores and Z-scores. MAS assesses trait anxiety and reflects long-term anxiety tendencies. This may have had a persistent effect on functional connectivity in the brain as repeated anxiety became stronger, resulting in lower functional connectivity as the MAS scores increased.

In the present study, the functional connectivity between brain regions in the right cerebral hemisphere was reduced in both psychological tests. The human brain is anatomically asymmetrical, with the left and right cerebral hemispheres specializing in different functions. Notably, the right cerebral hemisphere is characterized by more advanced emotional processing (Gainotti, 2012). Wang et al. (2022) reported that reduced activity in the right cerebral hemisphere is a critical component of anxiety that causes impaired negative emotional processing. Furthermore, Li et al. (2015) reported that patients with depression have a reduced saliency network in the right cerebral hemisphere. This suggests that abnormal functional connectivity in the right cerebral hemisphere is involved in anxiety. We can speculate that reduced functional connectivity occurred in brain regions located in the right cerebral hemisphere in the anxiety group.

In this study, we evaluated true anxiety by considering characteristic anxiety and the degree of anxiety. However, there was a decrease in the binding of the SN but not of the DMN.

This study has some limitations. First, the psychological test used was simplified and used only to assess the tendency toward anxiety. In this study, participants were not given auditory, visual, or other anxiety-provoking stimuli; however, they were evaluated for brain activity that occurs spontaneously in a resting state. Therefore, the differences in spontaneous brain activity between the normal and anxiety groups classified using the GHQ60 and MAS anxiety scales may not have been large enough to detect differences in functional brain connectivity. In the future, it is thought that changes in functional connectivity related to anxiety tendencies can be detected more clearly by using tasks to induce anxiety. Second, the difference in the number of people in the normal and anxiety groups was large, and the reliability of the data may not have been ensured. The large bias in the number of participants in the normal and anxiety groups may have contributed to the lack of stable results. These limitations may have contributed to the lack of significant functional connectivity differences between the normal and anxiety groups in networks other than the SN. In addition, the primary target population for this study was adolescents aged approximately 20 years. However, the age limit of the participants did not consider differences in anxiety characteristics among different age groups or changes in brain structure and function with age. It is difficult to generalize the relationship between anxiety and functional brain connectivity across different age groups. Therefore, in future studies, it will be necessary to replicate the results with a wider age range, a larger sample size, and equal numbers of participants in the normal and anxiety groups. Finally, it is impossible to deny the possibility that the MRI scan caused anxiety in the subjects. There are concerns that the anxiety and reactions to the MRI scan differed between the anxiety group and the normal group, and that this affected the functional connectivity of the brain. In the future, it will be necessary to evaluate the state anxiety of MRI scans and consider the impact of the imaging environment on psychological states.

## 5 Conclusion

This study used rs-fMRI to examine differences in functional brain connectivity at different degrees of anxiety among healthy participants. Notably, even within the healthy range, the functional connectivity within the SN was reduced in the high-anxiety group. This suggests that anxiety is involved in changes to the brain’s functional connectivity associated with emotion processing and cognitive control.

## Data Availability

The original contributions presented in the study are included in the article/supplementary material, further inquiries can be directed to the corresponding author.
